# Histamine: neural circuits and new medications

**DOI:** 10.1093/sleep/zsy183

**Published:** 2018-09-18

**Authors:** Thomas E Scammell, Alexander C Jackson, Nicholas P Franks, William Wisden, Yves Dauvilliers

**Affiliations:** 1 Department of Neurology, Beth Israel Deaconess Medical Center and Harvard Medical School, Boston, MA; 2 Department of Physiology and Neurobiology, University of Connecticut, Storrs, CT; 3 Department of Life Sciences and UK Dementia Research Institute, Imperial College London, UK; 4 Centre National de Référence Narcolepsie Hypersomnies, Unité des Troubles du Sommeil, Service de Neurologie, Hôpital Gui-de-Chauliac, Université Montpellier, INSERM, Montpellier, France

**Keywords:** histamine, H1R, H3R, pitolisant, diphenhydramine, narcolepsy, sedative, sleepiness, tuberomammillary nucleus, orexin

## Abstract

Histamine was first identified in the brain about 50 years ago, but only in the last few years have researchers gained an understanding of how it regulates sleep/wake behavior. We provide a translational overview of the histamine system, from basic research to new clinical trials demonstrating the usefulness of drugs that enhance histamine signaling. The tuberomammillary nucleus is the sole neuronal source of histamine in the brain, and like many of the arousal systems, histamine neurons diffusely innervate the cortex, thalamus, and other wake-promoting brain regions. Histamine has generally excitatory effects on target neurons, but paradoxically, histamine neurons may also release the inhibitory neurotransmitter GABA. New research demonstrates that activity in histamine neurons is essential for normal wakefulness, especially at specific circadian phases, and reducing activity in these neurons can produce sedation. The number of histamine neurons is increased in narcolepsy, but whether this affects brain levels of histamine is controversial. Of clinical importance, new compounds are becoming available that enhance histamine signaling, and clinical trials show that these medications reduce sleepiness and cataplexy in narcolepsy.

**Statement of Significance**

Histamine is a key wake-promoting neurotransmitter, and new medications that modulate histamine signaling are now under development. This paper reviews new research, ranging from basic science to recent clinical trials that highlight the normal functions of histamine neurons and how drugs that enhance histamine signaling may improve the symptoms of a variety of sleep disorders, including narcolepsy with cataplexy.

## Introduction

Towards the end of World War 1, an epidemic of encephalitis lethargica spread across Europe, and the Viennese neurologist Constantin von Economo observed that most patients with severe sleepiness had inflammation and lesions in the posterior hypothalamus (PH) [1]. He proposed that the PH contains wake-promoting neurons, and in the last decades, researchers have identified several, specific neuronal populations in the PH that are crucial for wake, including neurons producing histamine, orexin (hypocretin), GABA, and glutamate. The histaminergic system is now receiving increased attention as much has been learned in the last few years about the normal functions of this system and how its dysfunction may contribute to clinical sleep disorders [2, 3]. This article summarizes a symposium presented at the Sleep 2018 meeting focusing on the normal functions of the histamine system, how daily variations in activity of the histaminergic neurons help drive circadian rhythms of sleep and wake, and how new compounds that enhance histamine signaling improve wake and cataplexy in narcolepsy.

## Overview of Histamine Signaling

Histamine is a small, monoamine signaling molecule. Most clinicians are familiar with the functions of histamine in the periphery where it regulates immune responses and itch when released by mast cells and basophils, and how it regulates acid secretion when released by enterochromaffin-like cells of the stomach. However, in the brain, histamine mainly functions as a wake-promoting and rapid eye movement (REM) sleep–suppressing neurotransmitter, with additional effects on feeding and endocrine function [4].

**Figure 1.**
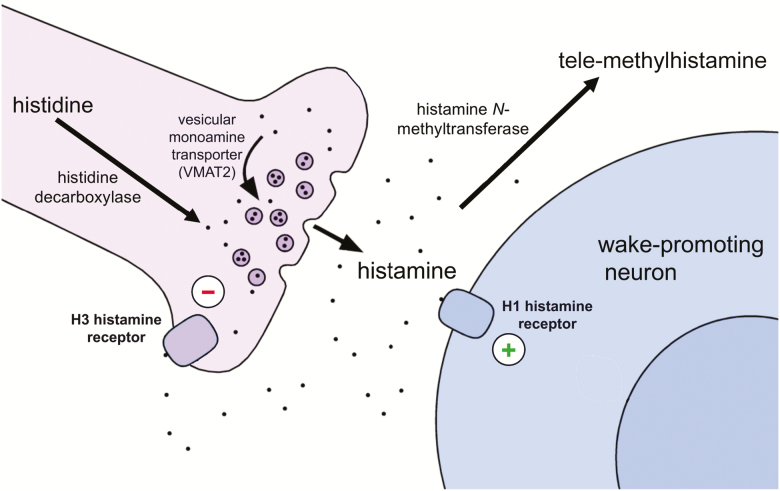
Histamine neurotransmission. Histamine is synthesized from histidine and then packaged into synaptic vesicles by vesicular monoamine transporter 2 (VMAT2). Upon release into the synaptic cleft, histamine can excite neurons via the H1 receptor leading to wakefulness, or it can inhibit histaminergic and other neurons via the H3 receptor. There is no reuptake mechanism for histamine, and it is broken down to tele-methylhistamine by histamine-N-methyltransferase.

In the brain, the tuberomammillary nucleus (TMN) is the sole neuronal source of histamine. The TMN is a loose constellation of 75–120000 neurons (in humans) scattered around the third ventricle and mammillary body in the ventral PH [5, 6]. The TMN neurons project widely throughout the brain, innervating and generally exciting neurons from the cortex to the brainstem. Histidine decarboxylase (HDC) converts the amino acid histidine to histamine which is then packaged into synaptic vesicles by the vesicular monoamine transporter (VMAT2) [7] (Figure 1). When nerve terminals are depolarized, histamine is released into the synaptic cleft and binds to histamine receptors which are located presynaptically or postsynaptically. In contrast to other monoaminergic neurotransmitters such as serotonin and dopamine, there appears to be no high-affinity reuptake system for histamine, but some histamine may be taken up by the low-affinity organic cation transporter 3 which is expressed by astrocytes [8]. Instead, most histamine is cleared from the extracellular space by conversion to the inactive tele-methylhistamine (tmHA) by histamine N-methyltransferase (HNMT) [4].

Histamine can act through four distinct G protein-coupled histamine receptors (H1-H4), and the H1, H2, and H3 receptors are all expressed in brain [4]. The H1 receptor depolarizes postsynaptic neurons and is crucial for the wake-promoting effects of histamine. In fact, over half of all over-the-counter sleep aids contain H1 receptor antagonists such as diphenhydramine or doxylamine. The H2 receptor may mediate aggression as mice with high histamine levels due to a lack of HNMT show more aggressive behaviors such as chasing and biting another male mouse, and these behaviors are strongly suppressed by a H2 receptor antagonist [9]. The H3 receptor is an inhibitory autoreceptor akin to 5HT1a or D2 receptors on serotonin- and dopamine-synthesizing neurons, respectively; when histamine tone is high, histamine can bind to the H3 receptor on TMN neurons, hyperpolarizing and reducing the activity of these cells [10]. The H3 receptor is also expressed as a heteroreceptor on a variety of neurons, including cells that make dopamine, serotonin, norepinephrine, acetylcholine, GABA, and glutamate. Thus, drugs that interfere with H3 receptor signaling such as pitolisant can block its inhibitory effects, increasing brain levels of histamine, as well as levels of serotonin, norepinephrine, dopamine, and possibly other neurotransmitters [11]. Strictly speaking, most H1 and H3 receptor “antagonists” are actually inverse agonists; H1 and H3 receptors are constitutively active, even in the absence of histamine, and drugs such as diphenhydramine and pitolisant reduce this constitutive activity.

## Histamine Signaling and Sleep/Wake Behavior

Much preclinical research demonstrates that histamine, acting via H1 and H3 receptors, is essential for normal sleep/wake behavior. TMN histamine neurons fire fastest during wake, especially active wake, and extracellular histamine levels are highest during the active period [12–14]. TMN neurons fire little during non-rapid eye movement (NREM) sleep, and they are essentially silent in REM sleep [12, 15–18]. Drugs that interfere with H3 receptor signaling (e.g. ciproxifan and pitolisant) promote wakefulness in mice, probably through an increase in levels of histamine and other wake-promoting neurotransmitters [2, 11, 19, 20]. Chemogenetic activation of the TMN neurons substantially increases locomotion, likely reflecting high levels of arousal [21].

Which systems activate TMN neurons during wake is unclear, but the orexin neurons are one important input. The orexin neurons heavily innervate and excite the TMN, and infusion of orexin-A into the TMN region increases wake [22–24]. Similarly, TMN neurons strongly express the OX2 orexin receptor, and focal restoration of this receptor in the TMN region improves maintenance of long wake bouts [25]. These observations demonstrate that the orexin neurons can activate the TMN, but surprisingly, histamine appears to have no effect on the orexin neurons [26].

Inhibition of histamine signaling or the TMN neurons has the opposite effects. Acute blockade of H1 receptor signaling by tiprolidine increases NREM sleep, and this effect is clearly mediated by the H1 receptor as this sedating effect is absent in mice lacking H1 receptors [19]. Acute optogenetic inhibition of HA neurons swiftly switches mice from wake to NREM sleep [27]. Similarly, mice with a mutation in the γ2 subunit of the GABA_A_ receptor are not sedated by zolpidem, one of the most heavily prescribed insomnia medications. However, when researchers restored the normal γ^2^ subunit only to the histamine neurons, zolpidem significantly reduced the latency to NREM sleep, demonstrating that an acute increase of GABAergic drive onto histamine neurons is sufficient to induce NREM sleep [28]. In addition, photoactivation of preoptic area GABAergic neuron fibers in the TMN induced sedation [29]. Collectively, these studies strongly suggest that histamine signaling promotes wake, and inhibition of histamine signaling promotes NREM sleep.

However, in contrast to the acute studies on histamine neurons, there are many studies demonstrating relatively normal sleep/wake behavior with chronic ablations of the different components of the histamine system. Rats with selective destruction of the TMN neurons have normal amounts of wakefulness when tested 1–2 weeks after the lesions [30]. In contrast to the acute effects of zolpidem on HA neurons mentioned above, mice with synaptic GABA_A_ receptors selectively removed from HA neurons have no altered sleep–wake characteristics, although they do take longer to fall asleep in a novel environment [31]. Similarly, HDC knockout mice which constitutively lack histamine or mice lacking H1 receptors have nearly normal amounts of wake [19, 32]. However, when mildly stressed by placement in a new cage, wild-type mice remain awake for 43 min, whereas mice lacking HDC stay awake for only 18 min [32]. HDC knockout mice are also significantly more drowsy at the start of the active period (“lights off”) compared with littermate controls [32]. These findings suggest that other arousal systems can partially compensate for a chronic absence of histamine signaling, but the necessity of histamine becomes clear when the mice are challenged to produce a high level of arousal. As acute pharmacological and chemogenetic manipulations of histamine signaling strongly affect sleep/wake behavior, it seems most likely that these subtle effects with chronic manipulations are reflective of the ability of other arousal systems to partially compensate for chronic reductions in histamine signaling.

Whether histamine and the TMN neurons affect REM sleep is less clear. Lesions of the TMN region produce a small but transient reduction in REM sleep [30], and ketotifen, a selective H1 receptor antagonist, suppresses REM sleep [33]. These studies suggest that histamine could promote REM sleep, but other research suggests the opposite. Hypothalamic neurons producing melanin-concentrating hormone (MCH) promote REM sleep [34, 35], and histamine inhibits the MCH neurons via H3 receptors [36]. Some MCH neurons also likely release GABA, and photoactivation of their terminals in the TMN elicits inhibitory postsynaptic responses in TMN neurons and lengthens bouts of REM sleep [37]. Similarly, mice lacking HDC have a small increase in REM sleep [32]. Our opinion is that histamine and the TMN neurons likely suppress REM sleep, but clearly, more work is needed to sort out the details.

Most research has focused on the TMN as a key source of histamine, but microglia can produce histamine in response to lipopolysaccharide [38], and about half of all brain histamine is produced by mast cells, which are scattered across the brain [39]. Asthma and allergic rhinitis are often worst at night, and the molecular circadian clock regulates the reactivity of mast cells in the periphery [40]. Much less is known about mast cells in the brain and what may trigger them to release histamine. One study found that activation of brain mast cells can increase wakefulness, and the sedating effects of selective H1 receptor antagonists such as tiprolidine are absent in mice lacking mast cells [41]. Mice normally show a strong increase in wake when food deprived, but mice without mast cells lack this response, either due to poor promotion of wake or reduced appetite [41]. In addition, chronic mild stress doubles the number of brain mast cells and increases wake during the active period [42]. Although these observations are intriguing, mice lacking mast cells have normal amounts of wake, NREM, and REM sleep [41], and determining how these cells contribute to normal or pathological sleep/wake behavior will be important directions for future research.

## Cellular Diversity and Function of Histaminergic Neurons

Although best known for their critical role in the regulation of the wake state and aspects of wakefulness such as motivation and cognition, TMN histamine neurons have also been implicated in a wide range of other physiological and behavioral functions, including thermoregulation, stress, and feeding [4]. Whether this functional diversity is attributable to molecular, cellular, and circuit-level heterogeneity is an unresolved question. Specifically, are histaminergic neurons a single, relatively homogenous, though multifunctional population, or do they consist of discrete subpopulations? Stable neuronal cell–type identity lies at the intersection of a variety of key features that include developmental lineage, morphology, intrinsic excitability, neurochemistry, and functional connectivity [43, 44]. Multiple lines of evidence suggest that histaminergic neurons may exhibit functional heterogeneity at several levels of analysis [45]. For example, different forms of stress have been shown to activate only selected populations of histaminergic neurons [46]. In other works, putative histaminergic neuron subpopulations exhibit differential sensitivity to GABA agonists, accounted for by divergent expression of GABA-A receptor subunits [47, 48]. Anatomical evidence suggests that subpopulations of histaminergic neurons coexpress neuropeptides such as galanin, substance P, and met-enkephalin in a species-dependent manner [49, 50]. As histamine neurons may take up L-DOPA and express DOPA decarboxylase, some have proposed that histamine neurons may have the capacity to synthesize and corelease dopamine [51]. Finally, studies using microdialysis in behaving animals have demonstrated projection target–dependent differences in histamine release in response to pharmacological manipulation of histaminergic neurons [52]. Considered together, these data suggest the existence of molecularly and functionally distinct subpopulations of histamine neurons.

Electrophysiological signatures can also distinguish functional subpopulations of neurons based on their biophysical properties [53]. The basic electrophysiological properties of histaminergic neurons are well described in a variety of species: low frequency, rhythmic firing, broad action potentials, and a long latency to fire after sustained hyperpolarization, largely explained by a prominent A-type potassium current [54–57]. However, electrophysiological diversity among histaminergic neurons is not well understood. Recently, Fujita and coworkers systematically analyzed the membrane properties of genetically defined histaminergic and nonhistaminergic neurons in the ventral TMN of adult mice followed by unsupervised hierarchical cluster analysis based on key electrical signatures [27]. This approach revealed a functional dichotomy among ventral TMN histamine neurons, with one putative subpopulation exhibiting higher input resistance, briefer action potentials, and higher maximal firing rates relative to the other subpopulation [27]. Although these data suggest that histamine neurons exhibit some functional diversity in their electrophysiological signatures, it remains an open question as to whether these putative subpopulations align with other molecular and circuit-level measures of diversity or represent a continuum of variation among these neurons.

Differential expression of genes can also shed light on histaminergic neuron diversity. Transcriptomic analysis at single cell resolution has emerged as a powerful and informative means of revealing cell-type diversity and circuit function in the brain. In particular, single cell RNA sequencing (scRNA-seq) allows the capture of mRNA from isolated single cells, and, through bioinformatic analysis, categorization of discrete cell types, even within complex brain structures such as the hypothalamus [58–62]. With regard to histaminergic neurons, two recent studies captured some histaminergic neurons as part of a larger scale census of the arcuate nucleus or whole hypothalamus [61, 62]. Preliminary single cell transcriptomic analysis of histaminergic neurons (Jackson and coworkers, unpublished data) has revealed that HDC-expressing neurons are highly distinct in comparison with other posterior hypothalamic neurons as they express a unique complement of both known and novel cell-type-specific markers. The histamine neurons robustly express transcripts for VMAT2 (*Slc18a2*) and glutamate decarboxylase 1 (*Gad1*), an enzyme that converts glutamate into GABA (see section on histamine and GABA co-transmission below). Further work will be required to determine the extent to which histaminergic neurons are transcriptionally homogenous or consist of distinct subpopulations. Furthermore, circadian time may be an additional variable as intriguing recent evidence suggests that *Hdc* expression varies across the day-night cycle [63]. In concert with an array of cell type-specific circuit-cracking methodologies, this approach is well suited to reveal the molecular basis for functional diversity in the histamine system.

## Histamine Production Is Governed by Mechanisms to Help Optimize Behavior With Circadian Time

A local circadian-like mechanism within histaminergic neurons helps optimize histamine levels for the times when animals will most likely be active. *Hdc* mRNA levels change rhythmically over 24 hr, and HDC protein levels peak during the dark when mice are most active [63]. This daily change gives rise to an increase in the apparent number of HDC-immunoreactive cells during the dark period [64]; during the light period, HDC-protein levels in some cells fall under the detection limit for immunocytochemistry. The rhythmic daily variation of *Hdc* gene expression is controlled by circadian transcription factors. Selective knock out of one such factor, the “master clock” *Bmal1* gene, from histaminergic neurons removes the oscillation of *Hdc* gene expression, producing higher and flatter levels of *Hdc* mRNA and HDC protein throughout the 24 hr [63]. Consequently, these mutant mice have abnormally high histamine levels during the “rest period,” resulting in sleep–wake fragmentation, with wakefulness intruding into sleep [63]. Most likely, circadian activity in TMN neurons is coordinated by the suprachiasmatic nucleus (SCN), but at present the communication route is unknown. This local circadian mechanism regulating histamine levels is one of many parallel mechanisms that could have evolved to ensure mouse behavior is optimized to the time of day.

## Histamine and GABA Cotransmission

Before histamine was identified as a brain neurotransmitter, it was already evident that there was a population of neurons in the TMN that innervated the neocortex. These cells were first proposed to be GABAergic as indicated by immunocytochemistry for the GABA-synthesizing enzyme GAD-67 and GABA itself [50, 65], and later, it was found that these neurons were also histaminergic [66, 67]. To be truly GABAergic, neurons also need to package GABA into synaptic vesicles, and two mechanisms are possible. The first is that VMAT2, which packages histamine, could also transport GABA as has been described in dopaminergic neurons of the ventral tegmental area, some of which corelease GABA [68, 69]. The second, more conventional mechanism, would be that GABA is packaged into vesicles via the vesicular GABA transporter, VGAT [70, 71], and some histaminergic neurons express the *Vgat (Slc32a1*) gene [21]. Because GABA and histamine are in separate vesicles, at least in cultured histamine neurons [72], it seems most likely that histamine neurons use VGAT to package GABA.

**Figure 2.**
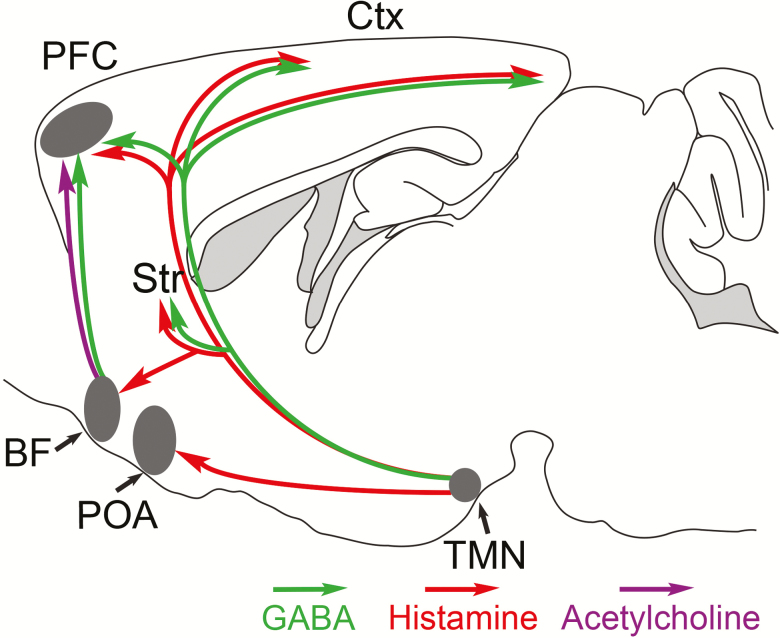
Schematic of circuitry for how Histamine–GABA neurons promote wakefulness. During wakefulness, histamine/GABA neurons release histamine (red) and GABA (green) into the prefrontal cortex (PFC), neocortex (Ctx), and striatum (Str). Histamine-only projections excite GABAergic neurons in the preoptic area (POA) that inhibit sleep-active GABA neurons. Modified from Ref. 93.

Functional evidence for GABA release was provided in a recent optogenetic study. Photostimulation of histaminergic axons in acute neocortex slices induced the release of GABA as indicated by activation of GABA_A_ receptors [21]. This GABA release occurred in the presence of histamine receptor antagonists and action potential blockers, indicating that there was no intermediate activation of GABAergic interneurons by histamine. So how is the GABA released from the histaminergic axons? Selective knockdown of *Vgat* gene expression from histamine neurons increased wake during the dark period and locomotion [21]. When *Vgat* expression from the TMN area was knocked out (not limited to the histamine neurons), photostimulation did not evoke GABA release in the cortical slices, suggesting that neurons in the TMN region, likely the histamine neurons, can release GABA in the cortex [21] (Figure 2).

The GABA released from histaminergic axons then binds to extrasynaptic GABA_A_ receptors to produce a tonic inhibition, which is a form of background GABA_A_ receptor inhibition conceptually similar in its effects to extrasynaptic metabotropic GABA_B_ receptor activation [21, 73]. What could be the role of GABA in this context? We tend to think of GABA signaling as promoting sleep and reducing cognition, but within the neocortex, GABA signaling is also essential for processing information during the wake state [74]. In this context, synaptic inhibition coming from particular classes of GABAergic neurons governs the precise timing of when excitatory pyramidal neurons fire. Similarly, it has been discovered from modeling studies that tonic inhibition might enhance cognition by augmenting the accuracy of action potential firing [75–77]. Through their broad innervation of the neocortex, TMN neurons may modulate network activity via histaminergic and GABAergic volume transmission. Currently, it is unknown if GABA and histamine are released from different zones of the axons or if this is coordinated, and not all histamine neurons are GABAergic [78]. Much more work is needed to flesh out this heterogeneity of histamine cells and what this means for this systems-level organization of the brain.

## Plasticity in the Histamine System

With disruption of other wake-promoting systems, the histaminergic TMN neurons are capable of remarkable adaptation. Narcolepsy type 1 (NT1) is characterized by a severe loss of orexin neurons, and two, independent, postmortem studies unexpectedly showed that the number of HDC-immunoreactive neurons is increased 64%–95% in the brains of people with NT1 [5, 6]. Normally, the orexin neurons strongly excite the TMN neurons [22], and loss of signaling from these cells may trigger compensatory changes in TMN neurons. The most straightforward explanation is increased expression of HDC, making TMN neurons easier to detect by immunostaining. In addition, orexin null mice show moderate increases in the numbers of HDC neurons [5], and zebrafish lacking a key enzyme for synthesis of catecholamines also show more HDC neurons [79]. However, this change in cell number is not seen in all orexin-deficient mouse lines nor in zebrafish lacking orexins [5, 6, 80]. Alternatively, it is possible that the increase in HDC neurons in NT1 is related to the process that kills the orexin neurons, but the specific factors that give rise to this plasticity and whether it has a physiological impact remain unknown [2].

## Histamine Is Not a Reliable Biomarker for Narcolepsy and Other Central Hypersomnias

Several studies have examined histamine and its main metabolite tmHA in clinical hypersomnolence disorders, but the results are controversial. Initial studies reported low cerebrospinal fluid (CSF) histamine levels in narcolepsy (both type 1 and type 2) and idiopathic hypersomnia [81, 82], and this general pattern was supported by another small study that lacked control subjects [83]. However, a subsequent larger study found no reduction in histamine or tmHA in a well-defined population of patients with narcolepsy and other hypersomnia disorders using a very sensitive assay (liquid chromatographic-electrospray/tandem mass spectrometric assay developed for the simultaneous analysis of histamine and its major metabolite tele-methylhistamine) [84]; furthermore, histamine levels did not correlate with subjective (Epworth Sleepiness Scale) and objective (Multiple Sleep Latency Test) measures of sleepiness or CSF orexin-A levels [85]. These results were in agreement with the normal cortical levels of *tele*-methyl histamine in orexin-/-mice compared with wild-type mice [20]. In a small number of patients with narcolepsy, CSF was collected again several months after the first sample; yet, histamine and tmHA levels showed no consistent change [86]. Altogether these data suggest that despite the increased number of HDC cells, extracellular histamine levels are likely normal in patients with narcolepsy.

Histamine is a labile molecule and is present only at very low (pM) concentrations in lumbar CSF, and future studies of histamine tone may require sampling of extracellular fluid in the brain itself, closer to the sites of histamine release. Thus, although the number of histaminergic neurons is increased in type 1 narcolepsy, our opinion is that histamine and tmHA in lumbar CSF are not useful biomarkers of narcolepsy or other centrally mediated hypersomnia disorders.

## Treating Sleep Disorders With Medications That Target Histamine Receptors

Signaling through the H1 receptor promotes wake, and drugs that block the H1 receptor are the most commonly used medications for insomnia. First-generation H1 receptor antagonists such as diphenhydramine, chlorpheniramine, and doxylamine penetrate the brain well due to their lipophilicity, producing sedation, whereas second-generation medications such as fexofenadine and loratadine are less lipophilic and much less sedating [87]. Some antidepressants and antipsychotics such as doxepin, amitriptyline, and olanzapine are also H1-receptor blockers with beneficial effect on insomnia.

The H3 receptor has become a recent drug target for the treatment of daytime sleepiness in narcolepsy and other hypersomnia disorders [10, 88]. Pitolisant (previously known as tiprolisant) is a H3-receptor inverse agonist that increases brain levels of histamine and other wake-promoting neurotransmitters [89, 90]. In mice lacking orexin, pitolisant increased wakefulness, decreased NREM sleep, and reduced cataplexy [20]. In a medium size, class 1 study of patients with narcolepsy (type 1 and type 2), pitolisant at doses ranging from 10 to 40 mg/day over 8 weeks reduced the Epworth sleepiness score about 6 points, a reduction similar to modafinil 100–400 mg/day [91]. Another recent study found that pitolisant 10–40 mg/day (mostly at 40 mg/day in this flexible dosing, 7 week study) reduced the weekly cataplexy rate by about 75% [92]. Pitolisant is generally well-tolerated, with only few adverse events that include headache, irritability, nausea, and insomnia. The starting daily dose should be 10 mg and it may be increased up to 40 mg, given once each morning. Pitolisant was approved by the European Medicines Agency in 2016 to treat adults with narcolepsy with or without cataplexy, and it will soon be reviewed by the FDA for use in the United States. Most research on H3 antagonists has focused on their use in narcolepsy, but they may improve sleepiness in disorders such as idiopathic hypersomnia, Parkinson’ disease, and obstructive sleep apnea syndrome, and clinical trials are ongoing.

How H3-receptor inverse agonists such as pitolisant promote wake and suppress cataplexy requires further research. Most likely, pitolisant promotes wake by increasing histamine tone, but as H3 receptors are expressed on other monoamine neurons, increases in norepinephrine, serotonin, and dopamine may also contribute [2, 11, 19, 20]. As histamine itself has little effect on REM sleep, it seems most likely that pitolisant suppresses cataplexy through H3 receptors on noradrenergic or serotonergic neurons that strongly suppress REM sleep.

Over the last decades, researchers have learned much about histamine’s effects on wakefulness and sleep and the effects of drugs that enhance signaling through the H1 and H3 histamine receptors. Still, important questions remain such as whether histamine is made by more than one type of neuron, what aspects of wakefulness are enhanced by histamine, are cotransmitters such as GABA important, what is the cause and the functional impact of the increase in histaminergic neurons in narcolepsy, what are the interactions between the histaminergic and orexinergic neurons, and what is the optimal use of H1 and H3 receptor antagonists in narcolepsy and other clinical disorders? With a more refined molecular, cellular, and circuit-level understanding of the function of the histaminergic system, we will ultimately be better positioned to deliver more effective and specific therapies for patients with sleep disorders.

## Funding

This work was supported by the National Institutes of Health (P01 HL095491 and R01 NS106032 to T.E.S; K99/R00 MH097792 and R01 MH112739 to A.C.J.); the UK Dementia Research Institute which receives its funding from UK DRI Ltd funded by the UK Medical Research Council, Alzheimer’s Society, and Alzheimer’s Research UK (N.P.F. and W.W.); and the Wellcome Trust (107839/Z/15/Z to N.P.F. and 107841/Z/15/Z to W.W).

*Conflict of interest statement*. T.E.S. has consulted with Bioprojet and Harmony Biosciences (makers of pitolisant). Y.D. has consulted for UCB Pharma, Jazz, Theranexus, Flamel, Idorsia, Takeda, Harmony Biosciences, and Bioprojet. A.C.J., N.P.F., and W.W. have no financial disclosures.
